# Novel stem cell and gene therapy in diabetic retinopathy, age related macular degeneration, and retinitis pigmentosa

**DOI:** 10.1186/s40942-019-0158-y

**Published:** 2019-02-13

**Authors:** Parker E. Ludwig, S. Caleb Freeman, Adam C. Janot

**Affiliations:** 1 Creighton University School of Medicine, 2500 California Plaza, Omaha, NE 68178 USA; 2 Vitreoretinal Institute, 7698 Goodwood Blvd, Baton Rouge, LA 70806 USA; 3 Department of Ophthalmology, Louisiana State University Health Sciences Center, New Orleans, LA USA

## Abstract

Degenerative retinal disease leads to significant visual morbidity worldwide. Diabetic retinopathy and macular degeneration are leading causes of blindness in the developed world. While current therapies for these diseases slow disease progression, stem cell and gene therapy may also reverse the effects of these, and other, degenerative retinal conditions. Novel therapies being investigated include the use of various types of stem cells in the regeneration of atrophic or damaged retinal tissue, the prolonged administration of neurotrophic factors and/or drug delivery, immunomodulation, as well as the replacement of mutant genes, and immunomodulation through viral vector delivery. This review will update the reader on aspects of stem cell and gene therapy in diabetic retinopathy, age-related macular degeneration, retinitis pigmentosa and other less common inherited retinal dystrophies. These therapies include the use of adeno-associated viral vector-based therapies for treatment of various types of retinitis pigmentosa and dry age-related macular degeneration. Other potential therapies reviewed include the use of mesenchymal stem cells in local immunomodulation, and the use of stem cells in generating structures like three-dimensional retinal sheets for transplantation into degenerative retinas. Finally, aspects of stem cell and gene therapy in diabetic retinopathy, age-related macular degeneration, retinitis pigmentosa, and other less common inherited retinal dystrophies will be reviewed.

## Background

Degenerative retinal disease afflicts many around the world and can lead to blindness. Age related macular degeneration is the leading cause of blindness in Caucasians greater than 40 years of age in the USA [1]. Diabetic retinopathy is the leading cause of vision loss in those between the ages of 20 and 74 [2]. Retinitis pigmentosa affects 1 in 3000–7000 people, making it one of the most common causes of inherited retinal disease leading to blindness [3, 4].

Current FDA (Food and Drug Administration)-approved treatment for neovascular age-related macular degeneration (AMD) and complications associated with diabetic retinopathy involve frequent anti-vascular endothelial growth factor (VEGF) intravitreal injections. Similarly, diabetic retinopathy is treated with anti-VEGFs and laser photocoagulation. Though effective in treating the complications associated with these diseases, they do little to reverse the course. Until recently, treatment for retinitis pigmentosa (RP) has consisted of measures to reduce further damage or slow the disease. However, FDA approval has been received of the gene therapy Luxturna (voretigene neparvovec-rzyl), which targets RPE65 [5–7].

Stem cell and gene therapy may also reverse the effects of these degenerative retinal conditions. Efforts have been made to develop novel therapies involving the regeneration of atrophic or damaged retinal tissue, prolonged administration of neurotrophic factors and/or drug delivery, immunomodulation, replacement of mutant genes, and immunomodulation through viral vector delivery. The purpose of this review is to introduce the retinal conditions and diseases most prevalent in patient populations, and to explore some of the novel treatment approaches currently under investigation; these include the use of stem cells and gene therapy techniques.

## Stem cells

While there is ambiguity in the definitions suggested, stem cells are generally identified as populations of cells that are both self-renewing, and capable of differentiating into multiple cell types, thus receiving the description of multipotent or pluripotent, depending on the situation [8]. It had been thought that the mature retina of mammals is incapable of regeneration; however, reports have shown that there are a population of retinal stem cells localized to the pigmented ciliary margin that are capable of differentiating into several types of retinal cells such as rod photoreceptors, bipolar cells, and Müller cells [9–11]. This population of cells has since been described as late-stage neuronal progenitors or pigmented ciliary epithelial cells [12, 13]. Neural progenitor/stem cells are important to retinal development, as the retina is a specialized appendage of the nervous system.

Among the types of stem or progenitor cells, identified by source, are human embryonic stem cells (hESCs), bone marrow stromal cells (BMSCs), human mesenchymal stem cells (hMSCs), human pluripotent stem cells (hPSCs), and human retinal progenitor cells (hRPCs). hESCs are derived from the transfer of preimplantation embryo cells into culture, and are classified as a type of hPSC along with human induced pluripotent stem cells; these cell lines maintain pluripotency until being differentiated, and were among the first progenitor cells used in regenerative research [14, 15]. hMSCs can differentiate into the various mesenchymal tissues such as osteoblasts, chondrocytes, and adipocytes. There is disagreement over the appropriateness of terms such as mesenchymal stem cell, and the related terms bone marrow stromal cell, mesenchymal progenitor cell, and bone marrow progenitor cell; hMSCs are generally understood to refer to the fibroblast-like cells shown, more recently, to also be capable of differentiating into non-mesenchymal lineages such as cardiac, renal, hepatic, and neural cells [16]. They are important to the normal function of hematopoietic stem cells, and have been investigated for use in cancer therapy due to their tendency to localize to solid tumors [17]. Sources for deriving hRPCs include fetal retinas, ESCs, and induced pluripotent stem cells (iPSCs); there is suggestion in the literature that the fetal-derived RPCs may be more suitable for therapy due to lower immunogenicity and increased stability [18].

The multipotent characteristic of the progenitor cells is important to the proper development of the structures in the eye and retina, and for this reason many have thought that there could be regenerative potential in the damaged or degenerate retina through the transplantation or activation of stem cells. Initial attempts at transplanting neural and retinal stem cells into the degenerating retina proved unsuccessful, and it was seen that these cells neither integrated into the retina, nor restored vision [19–22]. However, MacLaren et al. [23] noted that the mammalian retina can incorporate rod photoreceptor precursors derived from the post-natal day 1 (P1) retina of mice into the outer nuclear layer (ONL). They observed that these cells differentiated, formed functional synapses, and improved vision in mouse models of degenerative retinal disease. The conclusion drawn was that the ontogenetic stage of the precursor cells, defined in this case by the expression of neural retina leucine zipper (Nrl), is vital to the integration of the stem cells into the retina [23].

Others have observed that the transplantation of embryonic stem cell-derived neural progenitors leads to the enhanced survival of host retinal cells, such as photoreceptors, and improved preservation of visual function in the *mnd* mouse model of neuronal ceroid lipofuscinoses [24]. It has been suggested that this enhanced survival of host cells could be mediated by the secretion of growth factors, as IGF-1 administration has been seen to produce similar results [24–27].

Aharony et al. [28] investigated stem cells and their effects on ocular conditions. They organized the roles of stem cells in treatment of the eyes into three categories: vehicles for drug delivery, immunomodulatory agents, and mediators of tissue regeneration. Progress is being made in the understanding of each of these roles and the ways to effectively utilize them. In terms of tissue regeneration, researchers are often capable of inducing growth and development of transplanted cells, and in some cases, notable integration into the framework of the host cells is observed. In a study with primates treated with the hypoxia-inducing agent cobalt chloride and irradiation to mimic retinal disease, the transplantation of human embryonic stem cells (hESCs) was noted to result in survival and maturation of the transplanted cells, as well as some integration of the cells with host bipolar cells [29]. Singhal et al. [30] demonstrated that Müller glia which exhibit multipotentiality can be induced to differentiate into retinal ganglionic cells (RGCs) upon treatment with fibroblast growth factor 2 (FGF2) and γ-secretase inhibitor *N*-[*N*-(3,5-difluorophenacetyl)-l-alanyl]-*S*-phenylglycine *t*-butyl ester (DAPT) to inhibit Notch signaling. This differentiation into RGCs correlated to a significant improvement in retinal function as measured by electroretinogram.

Some studies have attempted to prime stem cells with environmental factors such as epidermal growth factor (EGF) prior to their transplantation in an effort to improve their incorporation into the host [31]. However, stem cells have been noted to migrate to the retina and differentiate into glia and ganglion cells in the absence of priming before transplantation when injected intravitreally or subretinally [28, 31]. Canola et al. [31] observed that this cell incorporation occurred to a greater extent in more advanced stages of disease. Importantly, few transplanted cells expressed photoreceptor markers, which may indicate that priming prior to or along with transplantation is necessary for the differentiation of photoreceptor lineages [28].

Bone marrow-derived stem cells (BMSCs) can differentiate into ganglion cells when administered intravitreally and intravenously following optic nerve injury in mice [28, 32, 33]. This differentiation effect was increased by administration of neuronal growth factors along with the cells [33].

Zhou and Xia [34] observed that transplantation of retinal stem cells in a state of glaucoma reduced levels of IFN-γ in both the serum and the aqueous humor which led to a decrease in inflammation. On the other hand, it has also been noted that in vitro study of mesenchymal stem cells (MSCs) in a rat retina-explant model show differentiation into microglia, which would induce inflammation rather than reducing it [28, 35].

Ischemia-associated retinal degeneration is a major cause of vision loss; Mathew et al. [36] found that intravitreal administration of BMSCs aided the survival of the retina in rats suffering ischemic damage. BMSCs had an anti-apoptotic effect through decreased TUNEL and caspase-3 expression, attenuated inflammation by reducing levels of TNF-α, IL-1β, and IL-6, and preserved autophagy. The transmembrane glycoprotein Prominin-1 (Prom1) has been shown to be an important regulator of autophagy in the RPE [37]. Understanding this role will aid in anticipating and controlling tumorigenesis in stem cell therapy.

One of the barriers to stem cell transplantation therapy is the difficulty of inducing incorporation of transplanted cells into the host cell structure; this could be complicated by immune reaction. Chao et al. [38] observed that human ESC-derived retinal neurons injected into the submacular space of a squirrel monkey continued to survive 3 months following the injection, and that some donor cells integrated into the host retina while some axons from donor cells extended into the optic nerve within the same time period.

Wahlin et al. [39] recently discussed a method for differentiating human pluripotent stem cells (hPSCs) into three dimensional (3D) retina models that bear many similarities to mature retinas. These similarities include structural outgrowth of resembling the outer segment of photoreceptors, neurotransmitter expression, and synaptic vesicle fusion. It is suggested that this model could aid researchers in studying the retina and the effects of various therapies on it in vitro. 3D retinal sheets generated using stem cells are being investigated for their ability to be transplanted into retinal degenerative mice [40, 41].

While inflammation can cause significant tissue damage, indications show that there is a link between inflammation and retina regeneration. The Xenopus genus of frog and other amphibians have the interesting ability to regenerate the whole retina upon its removal through activation of RPE cells to adopt multipotent characteristics [42]. Naitoh et al. [43] observed that the upregulation of matrix metalloproteinases through inflammatory cytokine upregulation was vital to retinal regeneration as dexamethasone or Withaferin A administration significantly suppressed RPE cell migration and transdifferentiation in the African clawed frog (*Xenopus laevis*).

Qu et al. [44] investigated the effects of combined human mesenchymal stem cell (hMSC) and human retinal progenitor cell (hRPC) subretinal transplant in rats. It was observed that the combined therapy resulted in improved electroretinogram performance, improved outer nuclear layer thickness, increased migration of grafted cells, and reduced activation of microglia and Müller cell gliosis as compared to single transplantation of hRPCs or hMSCs. Both MSCs and RPCs have been transplanted clinically for treatment of retinal disease, but this study suggests that a more optimal result may be achieved through combination therapy with both types of cells.

While stem cell transplantation has not yet developed into the unhindered, regenerative solution to all degenerative conditions as was initially hoped, much research is currently ongoing. Our understanding of stem cell capabilities and the varying modes that may be used therapeutically continues to progress (Table 1). Other applications will likely materialize into beneficial therapies following continued investigation.

**Table 1:** Stem cell therapy

Condition	Technique	Result
Diabetic retinopathy	Mesenchymal stem cells	Absorption of ROS through expression of sulfoxide reductase A
	Endothelial progenitor cells	Incorporation into host retinal tissue and prevention of neovascularization
Macular degeneration	Induction of pluripotent stem cells	Differentiation into photoreceptors and RPE cells and integration into the host cell structure
	Subretinal ESC transplantation	Regenerative therapy
Retinitis pigmentosa	Treatment with brain-derived neurotrophic factor	Improved survival of neurons and retinal ganglionic cells and preserves structure of the optic nerve
	Induction of neural stem cell secretion of ciliary neurotrophic factor	Protection of photoreceptor cells

## Gene therapy

**Fig. 1:**
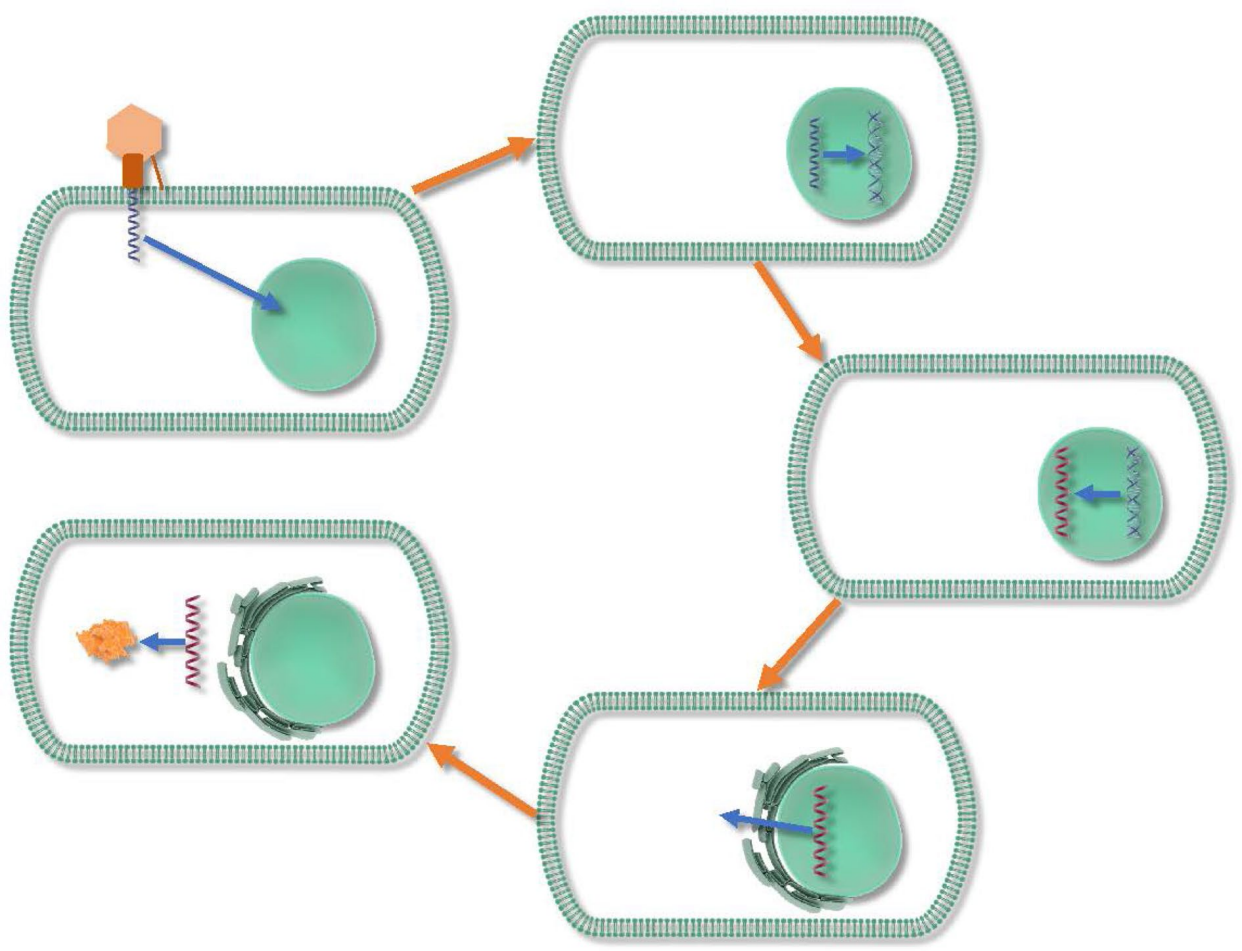
The general process of gene transduction with a viral vector: the AAV or other viral vector inserts its single-stranded DNA into the targeted cell, and the DNA is taken up into the nucleus where it is converted into double-stranded DNA by host cell machinery. This gene, along with its accompanying promoter is inserted between inverted terminal repeats to form an episomal concatemer in the host cell nucleus. Normal transcription and translation processes take place to produce the protein product of interest. DNA is depicted by the blue coils, and RNA by red coils

Interest has grown in the potential of gene therapy, the delivery of nucleic acid polymers into host cells to treat underlying conditions, for retinal diseases in recent decades (Fig. 1). The first demonstration of retroviruses acquiring cellular genes occurred in the mid-1970s, and this was followed by experimentation with retroviruses, simian virus 40 (SV40), bovine papilloma virus (BPV), vaccinia, and herpes simplex virus (HSV) [45]. In the time since the first use of viral vectors, much experience has been gained with vectors such as measles, vaccinia, polio, reovirus, adenovirus, vesicular stomatitis virus (VSV), lentivirus, γ-retrovirus, HSV, and adeno-associated virus (AAV). AAV has emerged as a favored vector for direct gene delivery in vivo due to its lack of pathogenicity and ability to incorporate into a variety of tissues in a directed manner (Table 2); one of its primary drawbacks is a limitation of packaged genetic material to 4.7 kb [45].

**Table 2:** Gene therapy

Condition	Goal	Method
Diabetic retinopathy	Reduction of angiogenesis	Downregulation of VEGF via gene targeting of sFlt-1, Flt23k, and PEDF
	Reduction of oxidative stress	Vector-mediated delivery of superoxide dismutase
	Regulation of renin–angiotensin system	Targeting genes of ACE 2, Ang, or Mas receptor
Macular degeneration	Inhibition of angiogenesis and neovascularization	Binding of VEGF by proteins delivered by AAV2 vectors
		AAV2 vectors carrying sFLT-1
		Viral vectors carrying endostatin and angiostatin
Retinitis pigmentosa	Decreased loss of photoreceptors and preserved retinal function due to improved phagocytic function	Vector delivery of MERTK gene
	Increased cell survival	Increased expression of GDNF
	Restore proper expression of the RPGR gene	Production of the retinitis pigmentosa GTPase regulator gene (commonly mutated in X-linked retinitis pigmentosa)

Boye et al. [46] reviewed the literature, and noted that gene therapy was particularly promising for the treatment of ocular disease due to the accessibility, immune-privileged nature, and compartmentalization of the eye. Some of the primary obstacles to long-term gene delivery include DNA degradation and promoter inactivation; as a result, many treatment strategies focus on the implementation of controlled release systems, optimization of promoters to aid DNA stability, and the reduction of regions heavy in cytosine and guanine (CpG sequences) [47].

Ezati et al. [48] investigated the efficacy of reprogramming RPE cells through AAV vector transduction of retinal progenitor cell genes. Larger increases were observed in neonatal RPE cell cultures than in adult cell cultures in general. Analysis indicated that there was an 80-fold increase in expression of the stem cell marker SOX2 (sex determining region Y-box 2) in neonatal culture as compared to control, and a 12-fold increase in adult culture. The increases in gene expression of other genes was less dramatic with there being 3.8-fold and 2.5-fold overexpression of nestin, and 3-fold and 2.5-fold increases in PAX6 expression, in neonatal and adult cells respectively. The ability to induce de-differentiation or reprogramming of RPE cells could have important implications for the regeneration of the retina in a diseased state.

2017 was a year of advances in gene therapy. It saw the first Food and Drug Administration (FDA) approval of gene therapies for certain forms of acute lymphoblastic leukemia, large B cell lymphoma, and biallelic RPE65-associated retinal dystrophy [49]. The excitement surrounding gene therapy and its potential is evident from the abundance of start-up companies devoted solely to the research and development of specific therapies, and the many millions of dollars being invested into such ventures [45].

## Retinal diseases and novel therapies

### Diabetic retinopathy

Approximately 422 million people around the world have been diagnosed with diabetes [50]. It is estimated that approximately 35% of those with diabetes have DR. Proliferative diabetic retinopathy (PDR) is the most common vision-threatening condition in individuals with type 1 diabetes, and DME is the leading cause of vision loss in people with type 2 diabetes [2, 51, 52].

Diabetes leads to damage retinal vasculature. This damage results largely from the effects of hyperglycemia on the basement membrane, endothelium, and pericytes of the retinal blood vessels [53]. Some of these changes have been noted to be induced by activation of protein kinase C, increased formation of glycation products, activation of polyol pathway, oxidation, and inflammation [54–57]. These processes lead to microaneurysm formation, vascular leakage, capillary non-perfusion, and neovascularization [53]. The presence of neovascularization defines PDR. Ultimately, diabetic retinopathy and secondary retinal ischemia lead to neuroretinal damage through neurodegeneration, gliosis, and neuroinflammation [53].

Current treatments for diabetic retinopathy, beyond management of the diabetes and hyperglycemia, most often focus on the vascular aspects of the condition. Anti-VEGF treatments (bevacizumab, ranibizumab, and aflibercept) have proven effective in reducing the vision loss in patients with DR. Anti-VEGFs decrease retinal edema by mediating VEGF’s action on endothelial cells and their adjoining junctions [53]. Anti-VEGFs also cause regression of neovascularization in PDR [58]. Panretinal photocoagulation and focal retinal photocoagulation are also used to effectively treat PDR and DME respectively [59–62].

Mesenchymal stem cells (MSCs) demonstrate potential as immunomodulatory agents in DR [63]. Through expression of sulfoxide reductase A, MSCs have been shown to absorb reactive oxygen species [64]. MSCs have demonstrated neuroprotective effects in animal models of retinal degeneration, and light- and ischemia-damaged retinas [65–67]. Endothelial progenitor cells (EPCs) have displayed some ability to repair damage in ischemic and diabetic retinopathy, though increased inflammation has also been reported; it appears that the EPC subtype, endothelial colony forming cells (ECFCs), are the cells capable of incorporating into the host retinal tissue and preventing neovascularization [63, 68, 69]. EPC deficiencies in diabetic animal models exhibit some improvement with the administration of modulating agents of granulocyte colony-stimulating factor (G-CSF), stromal cell-derived factor 1 (SDF-1), and some peroxisome proliferator-activated receptors (PPAR), as well as the drugs rosiglitazone, and atorvastatin [63, 70–75].

Wang et al. [76] recently suggested that gene therapy investigations into DR either focus on targeting existing neovascularization and vascular hyperpermeability, or protecting nerves and vessels from damage. Several studies have demonstrated an ability to downregulate VEGF including therapies targeting sFlt-1, Flt23k, and PEDF (which also decreased expression of matrix metalloproteinase and connective tissue growth factor) [77–82]. Other transgenes that have been targeted to reduce angiogenesis include endostatin, angiostatin, and tissue inhibitor metalloproteinase-3 [82–85].

Adhi et al. [86] observed that soluble cluster of differentiation 59 (sCD59) protected retinal neurons and the blood-retinal barrier from membrane attack complex-mediated damage. Other studies have aimed to increase neurotrophic factors like brain-derived neurotrophic factor (BDNF), decrease oxidative stress through manganese-dependent superoxide dismutase (MnSOD) delivery, or regulate the renin–angiotensin system with angiotensin-converting enzyme 2, Ang- [1–7], and the Mas receptor [87–92]. While therapies targeting each of the transgenes mentioned have shown efficacy in reducing or preventing damage in animal models of DR-related disease, there remains further investigation necessary into long-term efficacy and safety in humans.

### Macular degeneration

Age-related macular degeneration (AMD) is a disease of neurosensory retina and retinal pigment epithelium (RPE). AMD accounts for nearly 9% of worldwide blindness, and has become the leading cause of legal blindness in individuals over age 65 in the United States, Australia, Japan, and western Europe [93, 94]. In 2010, approximately 2.07 million people in the United States had AMD, which was up from 1.75 million in 2000 [95, 96]. AMD has a markedly increased prevalence in whites when compared to individuals of other ethnicities, with 1.85 million of the 2.07 million reported cases in 2010 being white [96]. It is estimated that 196 million people will have AMD globally in 2020, and 288 million in 2040 [93].

Presently, for dry AMD, the only treatment is oral administration of high-dose antioxidants (vitamins C and E, and beta carotene) and zinc which have been shown to slow the progression of AMD in a minority of patients [97]. Anti-VEGFs are the current standard of care in the treatment of wet AMD [98]. They lead to regression of choroidal neovascularization, prevention subretinal fibrosis, and decrease rates of severe vision loss in patients with wet AMD. Other options that are employed include laser therapy and photodynamic therapy targeting the choroidal neovascularization [99].

Induced pluripotent stem cells (iPSCs) have demonstrated similar effects to those previously discussed. The ability has been demonstrated to differentiate into photoreceptors and RPE cells and integrate into the host cell structure to significantly improve retinal function in retinal dystrophic and degenerative rat and mouse models [100–103]. It was also observed that these therapies were not accompanied by significant tumorigenesis which is important since rapid tumor formation is one of the primary concerns associated with iPSCs and ESCs [28]. Mandai et al. [104] transplanted an autologous iPSC-derived RPE cell sheet subretinally in a patient with advanced, wet AMD, and found that 1 year post-transplant, there was neither improvement nor worsening of visual acuity, and the graft remained intact. Cystoid macular edema was noted to be present. Further investigation is needed, but this study suggests that it could be feasible to transplant cell sheets to preserve remaining vision in degenerative diseases, but significantly improved results have not yet been demonstrated.

The initial studies of subretinal ESC transplantation in human patients with AMD and Stargardt disease indicated improved vision in the majority of patients on the order of 9–19 letters on the visual acuity test within a few months, and did not raise concern of serious safety issues in the small patient population thus treated [105, 106]. These studies suggest that stem cell transplantation has great promise as a potential regenerative therapy for individuals with AMD and Stargardt disease.

As mentioned previously, VEGF is a major mediator of angiogenesis in exudative AMD, and its inhibition can result in improved prognosis. VEGF antibodies and inhibitory agents have been used to good effect in the management of exudative AMD [46, 107, 108]. One of the inconveniences associated with VEGF antibody administration is that it requires regular intravitreal injections. AAV2 vectors carrying proteins that bind VEGF components have been effective in limiting new vessel formation in wet AMD. Extracellular VEGF is bound and prevented from interacting with the endothelial receptors FLT-1 and FLK by soluble FLT-1 (sFLT-1) [46]. Treatments with AAV2 vectors carrying sFLT-1 have inhibited neovascularization, and the use of rAAV.sFLT-1 and AAV2-sFLT01 have been shown to be safe in humans [109–112]. In phase 1 and phase 2a clinical trials, rAAV2-mediated gene delivery of sFLT-1 resulted in improved best corrected visual acuity (BCVA) in 62% of patients [111, 113, 114]. AAV2-sFLT01 is a novel chimeric protein that can be used to inhibit choroidal neovascularization (CNV). Pechan et al. [77] described the generation of two novel chimeric VEGF-binding proteins: sFLT01 and sFLT02. These proteins are comprised of an IgG-like chain of Flt-1 fused to either a human IgG1 Fc region or to its methyl domain. Several studies have confirmed the safety and efficacy of the AAV2 vector-mediated treatment of neovascularization in wet AMD models and patients [111, 112, 115–118]. Viral vectors carrying endostatin and angiostatin, the collagen and fibrinogen cleavage products, have efficacy in the inhibition of angiogenesis in exudative AMD [85, 119–121]. The safety of lentiviral Equine Infectious Anemia Virus (EIAV) as a method for ocular gene therapy in humans has been demonstrated in clinical trials [122]. Campochiaro et al. [122] noted that long-term expression of angiostatin and endostatin was observed through the latest measurement of eight neovascular AMD subjects after more than 2.5 years, and two subjects after more than 4 years. It was recently demonstrated that insulin-like growth factor binding protein-related protein 1 (IGFBP-rP1) inhibits retinal angiogenesis in mice with oxygen-induced retinopathy by blocking the extracellular signal-related kinase (ERK) signaling pathway and inhibiting VEGF expression [123].

The investigational drug HMR59 developed by Hemera Biosciences (Boston, Massachusetts, USA), uses an AAV2 vector to increase sCD59 expression; it aims to provide a therapy for dry AMD, and was granted ‘safe to proceed’ status by the FDA in January 2017 [124, 125].

### Retinitis pigmentosa and other inherited retinal dystrophies

Retinits pigmentosa (RP) is a group of inherited degenerative conditions that affect the photoreceptor cells of the retina. In the classic presentation of RP, the rods are preferentially targeted first, which leads to a loss of night vision and limits peripheral vision. As the disease progresses, central vision also becomes compromised resulting in legal blindness. The prevalence of RP globally is estimated at approximately 1 in 4000 [126, 127]. Depending on its form, RP can be inherited in an autosomal dominant, autosomal recessive, or x-linked recessive manner [126, 128–130]. Spontaneous mutations undoubtedly account for some cases of RP, as approximately 40% of RP cases are isolated instances that present without any other family members being affected [131]. Many mutations associated with RP have been identified [132–138].

Stem cells have been investigated as agents for prolonged administration of neuroprotective or neurotrophic factors. Aharony et al. [28] note that stem cells can be induced to secrete neurotrophic factors (NTFs) such as brain-derived neurotrophic factor (BDNF), ciliary neurotrophic factor (CNTF), glial cell-line-derived neurotrophic factor (GDNF), and vascular endothelial growth factor (VEGF) to treat degenerative ophthalmic conditions; however, it is mentioned that the effect of these agents administered through stem cell engraftment is currently suboptimal. Promising treatments do exist in this area: BDNF has been shown to improve survival of neurons and RGCs and to preserve the structure of the optic nerve [139–143]. CNTF-secreting NSCs have been noted to provide protection to photoreceptors cells in models of retinitis pigmentosa [144]. GDNF-secreting ESCs and BMSCs secreting a combination of GDNF, BDNF, and VEGF have also been shown to significantly improve RGC survival [28, 145, 146].

The mer receptor tyrosine kinase (MERTK) has received attention due to the involvement of its mutation in a very rare form of autosomal recessive retinitis pigmentosa [46, 147–149]. Retinal degeneration results from a subretinal accumulation of debris from the outer segment of photoreceptors due to inhibited phagocytic activity, and this leads to apoptotic photoreceptor loss and progressively worsening performance on eletroretinography [150–153]. Gene replacement studies targeting the MERTK gene have involved the vectors adeno-associated virus (AAV), adenovirus, and lentivirus [154–156]. Tschernutter et al. [156] used a lentivirus-mediated process, and observed improvement of phagocytic function, decreased loss of photoreceptors, and preserved retinal function for the 7 month examination period included in the published report. It has been shown that AAV-mediated CTNF expression suppresses electrophysiological retinal responses; however, AAV-mediated GDNF expression was not associated with the same adverse effects, but improved cell survival in combination therapy with lentivirus-mediated gene replacement [157]. The tyrosine-mutant AAV8 Y733F vector expressing a human MERTK cDNA driven by an RPE-selective promoter administered subretinally has been observed to improve retinal function in RP models for an 8 month study period, with improvement in phagocytic function, decreased retinal vascular degeneration, and inhibition of Müller cell activation being noted [158]. Interestingly, AAV8 vectors appear to exhibit a greater spread in a dog model than other AAV vectors such as AAV2; in a study conducted in a primate model, both vector types transduced RPE efficiently, but the AAV8 vectors were significantly better at targeting photoreceptors than the AAV2 vectors [159, 160]. Petit et al. [161] observed that the developmental stage of rods has an effect on the gene transfer efficiency of AAV vectors, suggesting that the ability of AAV vectors to infect dying rod cells could be limited, and the gene transfer efficiency markedly reduced. Following subretinal injection, subjects with altered development of rod outer segments exhibited significantly reduced AAV transduction of rods, and increased preference for cones. A preference for rods was observed when cells had matured. This type of increase in cone transduction was also observed in adult mice with retinal degeneration as compared to wild-type mice. An understanding of vector preferences to photoreceptors will aid researchers in developing effective delivery systems and treatments.

One of the major causes of X-linked retinitis pigmentosa (XLRP) is mutation of the retinitis pigmentosa GTPase regulator (RPGR) gene [162–165]. There has been difficulty in producing AAV vectors with RPGR due to the relative instability of its sequence [166–170]. Fischer et al. [166] optimized the coding sequence of RPGR^ORF15^ in an effort to increase sequence stability, increase expression levels of the RPGR transgene, and to remove cryptic splice sites. They demonstrated production of an AAV8 vector that consistently produced the full-length, correct RPGR protein, which rescued the disease phenotype in animal models. The glutamylation pattern of the vector-derived RPGR and that of the wild-type protein were indistinguishable which indicates a lack of significant alteration to post-translational modification. Appropriate safety was demonstrated in mice [166].

There have also been efforts to treat autosomal dominant RP (ADRP) using gene therapy. ADRP can be caused by mutations in more than 20 genes. Boye et al. [46] note that mutations may take the form of haploinsufficiency, dominant negative gene product, or toxic gain-of-function. In haploinsufficiency, the product produced by a single wild-type allele is not sufficient to maintain normal function. In dominant negative gene product mutation, there is usually interference with the movement or assembly of the product. Toxic gain-of-function mutations result in products that exhibit direct toxicity to the cell. More than 100 dominant mutations in the RHO gene alone have been identified [46]. Gene therapy differs depending on the type of mutation. In haploinsufficiency, therapy has involved delivery of wild-type cDNA to increase the level of normal protein to adequate levels such as in the treatment of retinal degeneration due to mutation in PRPH2 by Cai et al. [171]. Due to the complexity and diversity of dominant mutations, even those of the RHO gene alone, there have been efforts to treat the condition by using molecules such as ribozymes, synthetic miRNAs, or siRNAs to target and degrade both mutant and wild-type mRNA; due to the degenerative effect this has on the retina, these therapeutic agents must be accompanied by delivery and expression of cDNA resistant to degradation because of silent mutations [46, 172–179]. Some other approaches that exhibit promise in the treatment of ADRP include AAV vector delivery of wild-type cDNA to slow retinal degeneration, and the gene transfer of molecular chaperones to assist in proper protein folding or suppress the unfolding response [46, 180, 181].

There are many institutions, both private and academic, pursuing research into gene therapeutic agents to treat RP, XLRP, ADRP, and MERTK-related autosomal recessive RP [182]. Nightstar Therapeutics (London, England, UK) has an AAV vector encoding RPGR in phase I/II clinical trial [183]. The AAV2/5-hPDE6B vector from Horama (Paris, France) targets rod cGMP phosphodiesterase 6 β (PDE6B), and is in phase I/II clinical trial in patients with RP due to mutation of this gene [184]. MeiraGTx’s (New York, New York, USA) AAV2/5-hRKp.RPGR has also entered phase I/II clinical trial for the treatment of XLRP [185]. The RestroSense (acquired by Allergan in 2016) AAV vector RST-001 (Chop2) or ChR2 is intended primarily to treat retinitis pigmentosa, but advanced dry AMD is cited as a follow-on indication. It increases expression of the photosensitivity gene, channelrhodopsin-2 [186]. RST-001 is currently in phase I/II clinical trial for its application in advanced retinitis pigmentosa [187]. As an area of great interest currently, there are many investigations ongoing into gene therapies targeting various genes causing multiple types of retinal dystrophies.

In December 2017, the FDA approved Luxturna (voretigene neparvovec-rzyl, Spark Therapeutics. Philadelphia, Pennsylvania, USA) for the treatment of Leber’s congenital amaurosis type 2 and RP due to a mutation in RPE65 [7]. The therapy employs an AAV2 vector carrying complementary DNA (cDNA) encoding human RPE65. Voretigene neparvovec-rzyl is injected subretinally. The FDA indications for the drug require that the patient have a confirmed biallelic RPE65 mutation and viable retinal cells [188]. This drug is notable for being the first in vivo gene therapy approved by the FDA. In phase III clinical trial, it was demonstrated that patients receiving the drug experienced statistically significant improvements in multi-luminance mobility testing (MLMT), and full-field stimulus testing (FST) as compared to control, indicating restoration of RPE65 enzymatic activity resulting in improved navigation in low-to-moderate light and increased light perception. There were also slight improvements in best-corrected visual acuity (BCVA) noted. Ocular adverse events observed in the study participants were generally mild, with the most common being elevated intraocular pressure, cataract, retinal tear, and ocular inflammation [5]. Voretigene neparvovec appears to be a relatively safe therapy that could dramatically improve the vision of those with biallelic RPE65 mutation-associated retinal dystrophy.

## Future directions

Considering research into stem cells and their use in treating ocular conditions in recent years, it appears that their greatest utility is likely in environment modification through such practices as drug delivery and immunomodulation. The role of curing retinal diseases will be more directly addressed in the immediate future through gene modulatory therapies. Gene transduction through viral vector delivery is an area of aggressive research. While some of these novel treatments are already being used in clinical medicine, their continued potential for attenuating degenerative processes and improving vision is significant and deserves continued investigation. While there have been many mutations reported, there undoubtedly remain others yet undiscovered, and identification of genes and mutations amenable to targeted therapy should continue. AAV and lentiviral vectors are the staple of therapeutic gene delivery in retinal research, and while these techniques have yielded good results, it could be of benefit to further investigate the feasibility of retroviral transduction in conjunction with retinal stem cell proliferation induction or non-viral transfection techniques in retinal gene therapy. Non-viral gene therapy is another promising area of retinal research [189, 190]. Recent investigations have made encouraging progress and showcased remarkable potential therapies to improve the visual acuity and quality of life of millions of individuals around the world. The future of treatment in retinal disease likely lies in the utilization of some form of genetic modification to combat these blinding conditions.
